# High Levels of Erucic Acid Cause Lipid Deposition, Decreased Antioxidant and Immune Abilities via Inhibiting Lipid Catabolism and Increasing Lipogenesis in Black Carp (*Mylopharyngodon piceus*)

**DOI:** 10.3390/ani14142102

**Published:** 2024-07-18

**Authors:** Yan Liu, Dingfei Ma, Qiangwei Li, Leping Liu, Wenya Gao, Yuanyuan Xie, Chenglong Wu

**Affiliations:** National-Local Joint Engineering Laboratory of Aquatic Animal Genetic Breeding and Nutrition (Zhejiang), Huzhou University, 759 East 2nd Road, Huzhou 313000, China; dingfeima@outlook.com (D.M.); liqiangwei18@outlook.com (Q.L.); liuleping01@outlook.com (L.L.); yumianwen@outlook.com (W.G.); xieyuanyuan23@outlook.com (Y.X.)

**Keywords:** erucic acid, growth, lipid metabolism, lipid accumulation, antioxidative and immune capabilities

## Abstract

**Simple Summary:**

The erucic acid (EA) present in rapeseed oil is known to inhibit growth and lipid utilization in terrestrial animals, but its potential anti-nutritional effects on fish have remained unclear due to a lack of information. Therefore, we conducted this study to investigate the effects of varying levels of EA on growth, health, and lipid metabolism in black carp, *Mylopharyngodon piceus*. The results demonstrated that high-dose EA inhibited growth, induced lipid accumulation, reduced antioxidant and immune capabilities, and led to oxidative damage by suppressing lipid catabolism and increasing lipogenesis. This study fills a gap in our understanding of the physiological and anti-nutritional effects of EA on fish and provides valuable theoretical insights for enhancing the utilization of rapeseed oil in cultured carnivorous fish.

**Abstract:**

This study investigated the effects of dietary erucic acid (EA) on growth, lipid accumulation, antioxidant and immune abilities, and lipid metabolism in black carp fed six diets containing varying levels of EA (0.00%, 0.44%, 0.81%, 1.83%, 2.74%, and 3.49%), for 8 weeks. Results showed that fish fed the 3.49% EA diet exhibited lower weight gain, compared to those fed the 0.81% EA diet. In a dose-dependent manner, the serum triglycerides and total cholesterol were significantly elevated in the EA groups. The 1.83%, 2.74%, and 3.49% levels of EA increased alanine aminotransferase and aspartate aminotransferase activities, as well as decreased acid phosphatase and alkaline phosphatase values compared to the EA-deficient group. The hepatic catalase activity and transcriptional level were notably reduced, accompanied by increased hydrogen peroxide contents in the EA groups. Furthermore, dietary EA primarily increased the C22:1n-9 and C20:1n-9 levels, while decreasing the C18:0 and C18:1n-9 contents. In the EA groups, expressions of genes, including *hsl*, *cpt1a*, *cpt1b,* and *ppara* were downregulated, whereas the *fas* and *gpat* expressions were enhanced. Additionally, dietary EA elevated the mRNA level of *il-1β* and reduced the expression of *il-10*. Collectively, high levels of EA (2.74% and 3.49%) induced lipid accumulation, reduced antioxidative and immune abilities in black carp by inhibiting lipid catabolism and increasing lipogenesis. These findings provide valuable insights for optimizing the use of rapeseed oil rich in EA for black carp and other carnivorous fish species.

## 1. Introduction

Rapeseed oil (RO) is the third most abundant plant oil produced in the world after soybean oil and palm oil. It offers a high content of unsaturated fatty acids (FAs) and bioactive compounds to animals [1,2,3]. Due to its numerous health benefits, such as anti-inflammatory properties and the prevention of cardiovascular disease [4], RO has attracted extensive attention and is well known for being used as edible oil. In recent years, it has found an application in animal feed and appears to be an appealing choice as an alternative lipid source to fish oil because of its high output, low cost, and high nutritional value [2,4]. However, a growing body of studies based on years of practical application reveals that RO causes a series of adverse effects in farmed fish, including reduced growth, increased lipid deposition, decreased antioxidant properties, and impaired immune function [5,6,7,8,9]. These effects are detrimental to the health of farmed animals. In addition, feeding fish with RO has been reported to affect hepatic lipid metabolism in Atlantic salmon (*Salmo salar* L.) [10]. To maintain the sustainable use of RO in the feed industry, it is necessary to identify the causes of the negative effects caused by RO.

More and more research has shown that it is the erucic acid (EA) in RO that compromises the health of farmed animals [11,12,13,14]. EA is a 22-carbon long-chain monounsaturated omega-9 FA (C22:1n-9), widely distributed in the most important Brassicaceae food sources, and the highest EA contents are found in RO [15]. The level of EA in RO varies depending on the species and processing method, and the content can account for more than 40% in natural forms, but ranges from 0% to 54% in commercially proceeded RO. In earlier years, it was discovered that RO containing high levels of EA depressed growth and increased cardiac lipid contents in chicks and rats [12,14,16,17]. In addition, it decreased the digestibility and utilization efficiency of the diets [18]. Meanwhile, some in vitro studies verified that the adverse sides of the RO diet were closely associated with EA. Data from isolated rat hearts and livers showed that dietary EA impaired oxidative capacity and ATP production in mitochondria, thereby resulting in lipid deposition and hepatic steatosis [11,19]. Moreover, chronic feeding of a diet containing high-EA RO influenced the nutritional value of farmed animals, mainly altering individual fatty acid levels in tissues [16,20,21,22,23]. The physiological and anti-nutritional role of EA in fish is not well understood, as research on EA is limited and relatively recent. According to the latest research on grass carp, high concentration of EA could act as an anti-nutritional factor that was unfavorable for fish growth and even damaged immune function [13,24]. Therefore, based on the available studies, we hypothesize that the negative effects caused by RO may be related to the EA content.

Black carp (*Mylopharyngodon piceus*) is a typical carnivorous fish with high commercial value, being one of the most important aquaculture fish in China. The production of cultured black carp has been increasing rapidly year by year, accompanied by a growing demand for lipid sources. Additionally, fingerling black carp fed RO-based diets were found to accumulate increased lipid levels in the liver [9]. Therefore, this study chose black carp to examine the hypothesis that EA in RO induces the aforementioned negative effects. In the present study, six diets containing different concentrations of EA were fed to black carp for 8 weeks. Then, we assessed the effects of dietary EA on growth performance, lipid accumulation, antioxidant ability, and immunologic function. The results of this study will complement the existing knowledge of EA in fish physiology and anti-nutrition, providing some theoretical references for improving the utilization of rapeseed oil in cultured carnivorous fish.

## 2. Materials and Methods

### 2.1. Experimental Diets

Six experimental diets with varying levels of EA, all containing the same amount of nitrogen and lipids, were prepared at the National-Local Joint Engineering Laboratory of Aquatic Animal Genetic Breeding and Nutrition, located in Huzhou, China. Soybean oil and low EA RO (EA < 0.06%) served as the primary lipid sources of this study. Different amounts of EA (90% purity, Thermo Fisher, Norristown, PA, USA) (0.00%, 0.47%, 0.94%, 1.88%, 2.82%, and 3.50%) were designed, and different levels of palmitic acid (3.50%, 3.03%, 2.56%, 1.62%, 0.68%, and 0.00%) were added to maintain consistent lipid levels among the diet groups. In order to eliminate the influence of different lipid levels in diets on this experiment, the lipid levels were consistent among the groups (6.6%). The EA actual values detected in six diets were 0.00%, 0.44%, 0.81%, 1.83%, 2.74%, and 3.49%. The compositions of ingredients and FAs for the diets are presented in Table 1 and Table 2, respectively.

### 2.2. Feeding Experiment

Black carp juveniles were purchased from a local farm in Huzhou, China. They were then reared in experimental cages for two weeks to allow them to acclimate to the farming environment. Following the two-week acclimation period, 540 black carps (initial weight: 7.77 ± 0.02 g) were randomly assigned to 18 aquaculture net cages, with thirty fish per cage. The fish were fed three times daily, at 08:00, 12:00, and 16:00, with a feeding rate of 2% of their total body weight. The feeding experiment with the experimental diets was conducted for 8 weeks. During the entire feeding period, the water temperature was held within the range of 26–28 °C, while the pH was kept between 7.6 and 7.8. The dissolved oxygen content was maintained above 7.0 mg/L. 

### 2.3. Sample Collection

At the end of the feeding experiment, the total weight of fish in each cage was measured. From each group, 12 fish were randomly chosen and initially anesthetized using diluted tricaine methanesulfonate (Sigma, Livonia, MI, USA). Following anesthesia, data pertaining to their growth performance were meticulously recorded and subsequently calculated. A total of 8 fish were randomly chosen from 12 fish of each treatment group for collecting serum and liver samples. To obtain serum samples, blood was collected from the tail vein of the fish. The blood samples were then centrifuged at 3000 rpm for a period of 10 min. This process allowed for the separation of serum from the other components of the blood, which was then ready for further analysis or storage. Liver samples were used for the analysis of total lipid, FA profiles, metabolite contents, enzymatic activities, and mRNA levels. Another 5 fish (not from the above 12 fish), without having blood drawn, were selected for analyzing the whole body composition.

### 2.4. Experimental Data Collection

#### 2.4.1. Biochemical Analysis in Serum and Liver

The metabolite contents and enzymatic activities related to lipid metabolism (TG, TC, and GLU), antioxidation (MDA, CAT, T-SOD, T-AOC, and H_2_O_2_), and immunity (ACP and AKP) in both serum and liver were detected according to the instructions provided by the kits from Nanjing Jiancheng Bioengineering Institute, Nanjing, China.

#### 2.4.2. Lipid Content and Fatty Acid Composition 

The lipids in the diets and liver were extracted using the previously described separation method [25]. Subsequently, gas chromatography (Agilent 8890, Santa Clara, CA, USA) was used to measure the contents of individual FAs in the diets and liver. The analysis process and representation of the data from gas chromatography were consistent with those described in the previous literature [25,26].

#### 2.4.3. Analysis of Whole Body and Diet Composition

The fish samples, which had been cut to the appropriate size, were introduced into a vacuum drying oven (Marin Christ Alpha2-4 LSC Basic, Osterode, Germany). The purpose of this step was to remove moisture from the samples until they reached a stable, constant weight. The initial and final weights of the samples were recorded, and the moisture content was calculated based on the difference between these two weights. Subsequently, an electric furnace was employed to completely carbonize the dried samples. Following carbonization, the samples were transferred to a muffle furnace (Neytech 3-1750A, Torrance, CA, USA) and heated to 550 °C for a duration of 6 h. This process was conducted to determine the ash content of the samples. Finally, the crude protein content of the samples was analyzed utilizing the Dumas method, which was performed on an Elementar Rapid N exceed analyzer (Hanau, Germany). This method provided an accurate measurement of the protein content within the fish samples.

#### 2.4.4. Detection of the mRNA Levels of Lipid Metabolic, Antioxidative, and Inflammatory Genes

The following experimental processes were conducted: total RNA isolation (using Trizol, Takara, Dalian, China) and reverse transcription of RNA (using Takara reagents, Dalian, China) to obtain cDNA for real-time quantitative PCR (RT-qPCR). The RT-qPCR assay was performed on Bio-Red CFX96 Touch (BIO-RAD, Hercules, CA, USA) according to the following procedure: initial denaturation at 95 °C for 10 min, followed by 40 cycles of 95 °C for 15 s and 60 °C for 1 min. The primer sequences used in this study are listed in Table 3. The 2^–ΔΔCt^ method [27] was used to calculate the mRNA expression levels of the genes detected in this study. 

### 2.5. Statistical Analysis

A one-way analysis of variance (ANOVA) was used to analyze all the data in the present study using the SPSS software package, version 26.0 (Chicago, IL, USA). Tukey’s multiple range test was chosen for multiple comparisons among the experimental groups. All graphs were created using GraphPad Prism 9.0 (San Diego, CA, USA).

## 3. Results

### 3.1. Growth Parameters

The dietary EA had significant effects on FBW, WG, SGR, and CF (*p* < 0.05), but it did not affect VSI, HSI, MFI, and FCR (*p* > 0.05), as shown in Table 4. Fish fed the diet containing 0.81% EA exhibited higher FBW, WG, and SGR compared to those fed 3.49% EA (*p* < 0.05). Additionally, the CF value in the 0.00% EA group was significantly lower than in the other five groups (*p* < 0.05). The 2.74% and 3.49% diets significantly increased the whole body lipid levels and decreased their protein contents compared to those of the 0.00% and 0.44% diets (*p* < 0.05). No significant differences were observed in the values of whole body moisture and ash contents (*p* > 0.05).

### 3.2. Serum and Liver Biochemical Indicators

The effects of dietary EA on serum and liver biochemical indicators are summarized in Table 5 and Table 6, respectively. The 3.49% EA group had a higher total lipid level in the liver compared to the other experimental groups (*p* < 0.05). The serum and liver TG and H_2_O_2_ contents in the EA groups were elevated in a dose-dependent manner. The 0.00% EA group had a lower serum TG level compared with the other five groups, while higher liver TG values were observed in the 2.74% and 3.49 EA groups (*p* < 0.05). Meanwhile, the highest levels of H_2_O_2_ in serum and liver were found in the 2.74% group and the 3.49 EA group, respectively (*p* < 0.05). The dietary EA dramatically increased the total cholesterol (TC) contents in the serum and liver compared to the 0.00% EA diet (*p* < 0.05). Conversely, the serum GLU contents in the EA groups were significantly decreased with increasing EA levels (*p* < 0.05). The 0.00% and 0.44% EA groups showed higher GLU contents than the other four groups (*p* < 0.05).

The activities of CAT in the serum and liver were largely reduced in the EA groups (0.44–3.49%), all being lower than in the 0.00% EA group (*p* < 0.05). In the liver, T-SOD activities were decreased in the fish fed a 1.83% EA diet compared to the 0.00%, 0.44%, 0.81%, and 2.74% EA diets (*p* < 0.05). Additionally, the lowest T-AOC activity in the liver was found in the 3.49% EA group (*p* < 0.05). 

Significant differences in the MDA levels were found in the liver (*p* < 0.05), but not in the serum (*p* > 0.05), among the six groups. The 3.49% EA group exhibited the highest MDA value in the liver, which was significantly higher than that in the 0.44%, 0.81%, and 1.83% EA groups (*p* < 0.05). Furthermore, a 2.74% EA diet resulted in higher ALT and AST activities, compared to the 0.00%, 0.44%, 0.81%, and 1.83% EA diets (*p* < 0.05). When the level of dietary EA was at 2.74%, the ACP and AKP values in serum and liver were significantly decreased compared to 0.00% (*p* < 0.05).

### 3.3. Fatty Acid Contents

The effects of dietary EA on fatty acid composition in the liver are listed in Table 7. Dietary EA significantly increased the C22:1n-9 content in the liver, which was proportional to its concentration in the feed (*p* < 0.05). Fish fed with 2.74% and 3.49% EA diets exhibited the highest levels of C22:1n-9 compared to those fed the 0.00%, 0.44%, and 0.81% EA diets (*p* < 0.05). Additionally, the contents of C16:0, C16:1n-7, C18:2n-6, C18:3n-6, C20:1n-9, C20:4n-6, C14:0, and C22:5n-3 were elevated due to dietary EA (*p* < 0.05). In contrast, with increasing EA content in the diets, the C18:0 level was markedly decreased. The 2.74% and 3.49% groups showed the lowest percentage of C18:0, while the highest value was found in the 0.00% group (*p* < 0.05). Similar trends were observed in the contents of C18:1n-9, C22:0, and total MUFAs. The experimental groups had comparable percentages of C18:3n-3 (*p* > 0.05). However, when the level of dietary EA was 1.83%, fish showed the highest values in C22:6n-3, total PUFAs, total n-3 PUFAs, total n-6 PUFAs, and the n-3/n-6 ratio (*p* < 0.05).

### 3.4. mRNA Levels of Genes Relevant to Lipid Metabolism

**Figure 1:**
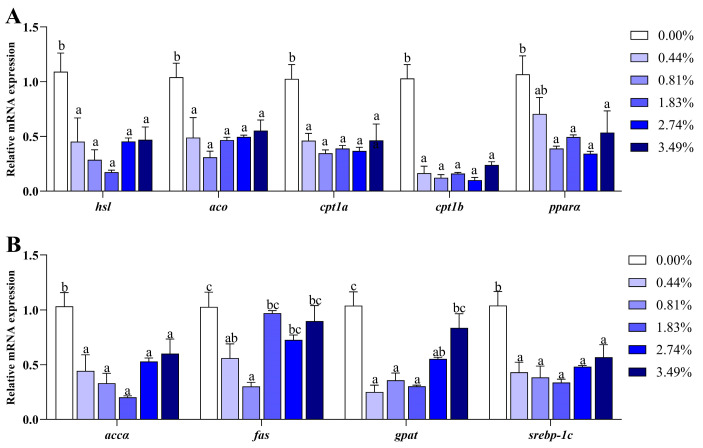
Effects of dietary EA on expressions of genes related to lipid metabolism in liver of black carp. (**A**) Lipid catabolism-related genes: *hsl*: hormone-sensitive triglyceride lipase; *aco*: acyl-CoA oxidase; *cpt1a*: carnitine palmitoyl transferase 1a; *cpt1b*: carnitine palmitoyl transferase 1b; *pparα*: proliferator-activated receptor α. (**B**) Lipogenesis-related genes: *accα*: acetyl-CoA carboxylase α; *fas*: fatty acid synthase; *gpat*: glycerol-3-phosphate acyltransferase; *srebp-1c*: sterol regulatory element-binding transcription factor 1c. Values are presented as means ± SEM (*n* = 8), and different superscript letters indicate significant differences (*p* < 0.05).

Dietary EA downregulated the expressions of genes involved in lipolysis (*hsl*), fatty acid beta-oxidation (*aco*, *cpt1a*, *cpt1b* and *pparα*), and lipogenesis (*accα* and *srebp-1c*) (*p* < 0.05) (Figure 1). There was an exception, as the 3.49% EA diet significantly increased the mRNA levels of *gpat* compared to the 0.44%, 0.81%, and 1.83% EA diets (*p* < 0.05). In addition, the lower expressions of *fas* were observed in the 0.00% and 0.44% groups (*p* < 0.05). 

### 3.5. mRNA Levels of Genes Involved in Antioxidative Capacity and Inflammation

**Figure 2:**
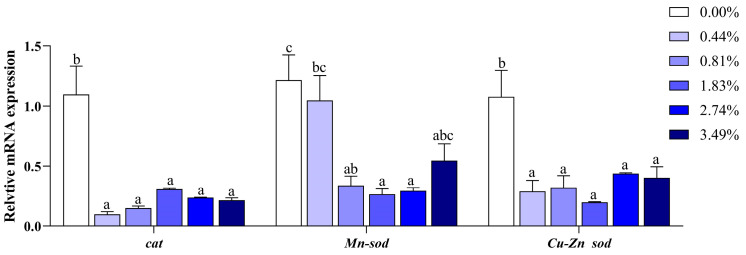
Effects of dietary EA on antioxidant-related genes in the liver of black carp: *cat*: catalase; *Mn-sod*: Mn-superoxide dismutase; *Cu-Zn sod*: Cu-Zn superoxide dismutase. Values are presented as means ± SEM (*n* = 8), and different superscript letters indicate significant differences (*p* < 0.05).

**Figure 3:**
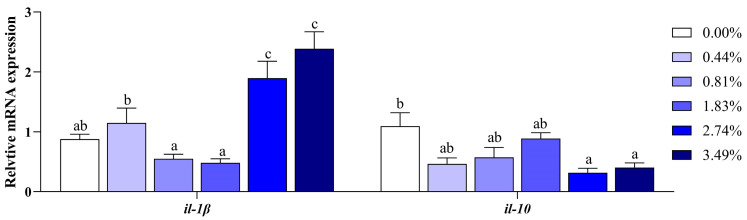
Effects of dietary EA on the expression levels of immune-related genes in the liver of black carp: *il-1β*: interleukin 1 beta; *il-10*: interleukin 10. Values are means ± SEM (*n* = 8), and different superscript letters indicate significant differences (*p* < 0.05).

When the dietary EA content was increased, it led to decreased transcriptional levels of genes related to antioxidant ability, specifically *cat*, *Mn-sod*, and *Cu/Zn sod* (*p* < 0.05) (Figure 2). The expression of *il-10* was reduced, particularly in the 2.74% and 3.49% EA groups, compared to the 0.00% EA group (*p* < 0.05) (Figure 3). However, the 2.74% and 3.49% EA induced higher expressions of *il-1β* compared to the other four groups (*p* < 0.05). 

## 4. Discussion

Increasing evidence suggests that the physiological side effects induced by RO diets are largely attributed to EA in terrestrial animals [11,12,13,14]. In aquatic animals, to date, only two studies on EA have been found in grass carp [13,24]. Considering the increasing use of RO in compound feeds, it still requires further investigation to determine the physiological and potential anti-nutritional effects of EA in aquatic animals. Early studies revealed that EA inhibited growth, which was observed in chicks [28] and rats [18]. Conversely, another study found that WG increased in rats fed diets containing EA (5.25%) [11]. Our study showed that as dietary EA levels increased, WG initially elevated from 83% to 93.53% and then declined to 70.6%. Feeding 0.81% EA resulted in a higher WG than 3.39% EA, and no significant differences were found among the other four groups. This suggests that a low concentration of EA did not affect growth, while a high concentration had an inhibitory effect, unlike the dose-dependent reduction reported in a previous study, where Gan et al. reported that even a small amount of EA (0.6%) led to a significant decrease in growth in grass carp [13]. In that study, grass carp were fed four times a day to visual satiation, but in the present study, the feeding rate of black carp was 2% of the total weight of fish, three times a day. This might explain the inconsistent results of EA on growth in black carp and grass carp, in addition to species differences. Our findings indicate that black carp is more tolerant to EA in their diets compared to grass carp, and that the effect of EA on growth depends on its content. 

Lipid deposition has been demonstrated to be one of the most common effects induced by long-term EA intake [11,16,22,23]. The current study also showed that dietary EA led to lipid accumulation, similar to reports in rats [11,22,23] and chicks [16]. Although HSI was not influenced, the total lipid level in the liver was significantly increased when fish were fed a 3.49% EA diet (Table 6). Most importantly, the TG and TC levels in the serum and liver were elevated in a dose-dependent manner. This aligns with the results of previous studies in rats and chicks [11,16], where increasing EA intake led to increased lipid contents. Our findings indicate that higher levels of EA impede lipid utilization and tend to promote lipid deposition. It is well known that carbohydrates and lipids are the main energy sources for animals, and they cooperate with each other to provide energy [29,30]. When the energy supply from one source is impaired, the other can serve as an alternative [29,30,31]. Therefore, the finding that the serum glucose levels decreased with increasing dietary EA indicates that EA enhanced glucose metabolism, thereby indirectly suggesting that lipid utilization was reduced in this study. Changes in the lipid content are often accompanied by alterations in fatty acid composition [25]. Results from terrestrial animals have confirmed that dietary EA affects fatty acid profiles in tissues [16,18,20,28,32,33]. This study revealed that hepatic EA deposition was significantly increased and directly proportional to the amount in the diet, in line with the findings from in vivo and in vitro tests on terrestrial animals [16,18,20,32,33]. The EA absorbed from the diet can be decomposed through oxidation into C20:1n-9 and C18:1n-9 [33,34,35]. Therefore, in our study, liver C20:1n-9 was elevated in fish fed a 2.74% EA diet. By contrast, instead of increasing, dietary EA resulted in a sharply reduced C18:1n-9 level. This implies that C18:1n-9 utilization for energy provision was enhanced, which might be a compensatory reaction to increased lipid deposition. More importantly, the absorbed EA in fish possibly tends to be oxidized into C20:1n-9 rather than C18:1n-9. This could be another reason why the C18:1n-9 level was not increased, as expected, but decreased. Additionally, prolonged intake of EA caused significant increases in long-chain FAs, including C16:0, C16:1n-7, C18:2n-6, C18:3n-6, C20:4n-6, and C22:5n-3 in black carp, similar to results in rat heart FA composition [20,22,23]. The retention of these FAs was likely due to impaired lipid oxidation, which could explain why excessive lipid deposition occurred. In summary, dietary EA led to lipid accumulation and alterations in FA profiles, with specific effects on the levels of various FAs in black carp. 

Accumulated liver lipids are largely due to dysregulated energy metabolism [11,16,23,30,31] and are predominantly caused by a general inhibition of lipid catabolism [11,31]. In rats, a few studies have shown that high doses of EA hinder energy supply from lipids through suppression of fatty acid beta-oxidation [6,10,11,19,28]. Meanwhile, very long-chain fatty acids are primarily oxidized in peroxisomes, with *aco* being a key enzyme in this process [36]. Data from rats indicated that EA promoted peroxisomal oxidation by upregulating *aco* [11]. In contrast to previous studies, fish fed with varying concentrations of EA (0.44%, 0.81%, 1.83%, 2.74%, and 3.49%) in this study, all exhibited lower expressions of *aco*. The increased C20:1n-9 levels observed in Table 7 indicated that the peroxisomal oxidation of EA was activated. Previous findings reported in tilapia and mammals have revealed that continuous peroxisomal oxidation tends to increase the risk of oxidative injury [31,37]. Consequently, in our study, the decreased *aco* expression may be a compensatory response to the increased oxidation capacity of FAs in peroxisomes [31]. Mitochondria are considered the main sites for energy production, and mitochondrial beta-oxidation is essential for controlling energy balance [38,39,40]. Previous studies have demonstrated that impaired mitochondrial beta-oxidation is responsible for increased lipid accumulation [11,17,19,28,40]. Our study found that EA caused significant down-regulation of *cpt1a* and *cpt1b*, which are the rate-limiting enzymes in mitochondrial beta-oxidation [40]. This suggests that the mitochondrial oxidation capacity was inhibited, which explains the increase in fatty acid contents, especially long-chain fatty acids (Table 7). Meanwhile, *pparα*, a key transcriptional regulator of genes involved in FA oxidation [41], was not activated and was also downregulated in the EA groups compared to the 0.00% EA group. This further suggested that FA beta-oxidation was reduced in the black carp fed EA diets. Furthermore, the mRNA level of *hsl* (crucial hydrolases of TG) in fish fed EA diets was significantly decreased, indicating that EA suppressed the hydrolysis of TG. On the other hand, lipogenesis plays a crucial role in maintaining lipid homeostasis, and it is closely associated with increased lipid accumulation [42]. In the present study, although the expressions of *srebp-1c* and *accα* were inhibited, the transcriptional levels of *fas* and *gpat* were elevated in the EA groups. These genes participate in the regulation of fatty acid de novo synthesis and TG synthesis [42]. Consequently, more TGs were accumulated in the liver of fish fed high levels of EA (2.74% and 3.49%) (Table 6). Collectively, dietary EA contributed to excessive lipid deposition in black carp by impairing lipid catabolism and increasing lipogenesis. 

Many studies have confirmed that excessive lipid deposition in the liver ultimately induces oxidative damage [5,7,13,21]. The oxidation-generated H_2_O_2_ has been reported to be a major source of oxidative stress in many studies [11,31], and the metabolism of EA remarkably caused an increase in the H_2_O_2_ levels [11]. This study showed that serum and liver H_2_O_2_ contents were both strongly elevated with increasing dietary EA levels. However, the CAT activity in the serum and liver was not increased but significantly decreased in the EA groups, which could break down hydrogen peroxide into water in order to reduce oxidative damage [43]. This explains why more H_2_O_2_ accumulated in the liver and serum. The SOD activity in the livers of fish fed 1.83% EA was decreased, suggesting its failure to prevent lipid peroxidation. Furthermore, our study showed that dietary EA downregulated the expressions of *cat*, *Mn-sod*, and *Cu-Zn sod*, in line with the results obtained in grass carp [24], further indicating that the dietary EA reduced the antioxidant capacity in black carp. Additionally, EA (2.74% and 3.49%) enhanced the liver MDA content compared to the 0.44% group. MDA, produced from lipid peroxidation, increased the risk of oxidative damage [44]. Finally, elevated serum ALT and AST activities were observed when the EA content exceeded 0.81%, indicating that liver injury had occurred. 

Oxidative damage is reported to cause an inflammatory response [5,9]. A previous study in grass carp demonstrated that EA in diets impaired the immune function by activating pro-inflammatory factors [24]. In this study, the upregulation of the pro-inflammatory factor *il-1β* in fish fed EA (2.74% and 3.49%) potentially contributed to the inflammatory response. The anti-inflammatory factor *il-10* could effectively inhibit inflammation. Previous studies found that an increased expression of *il-10* in crucian carp (*Carassius auratus*) and grass carp (*Ctenopharyngodon idella*) improved the immune ability of fish to resist bacterial infection [45,46]. When the fish were fed EA diets (0.00–1.83%), there were no significant differences in the mRNA levels of *il-10*, implying that a low-dose EA diet did not induce an inflammatory response in black carp. Nevertheless, the transcriptional levels of *il-10* in fish fed EA (2.74% and 3.49%) were largely decreased, indicating that the anti-inflammatory response was impaired. In addition, it is well-known that AKP and ACP activities are important indicators for assessing the health status of animals [47,48]. Adverse environmental conditions, such as oxidative stress, result in a significant decrease in AKP and ACP activities, and there is a significant increase in the expressions of inflammatory factors [49]. In our study, the continuous decrease in AKP and ACP activities in the serum and liver proved that EA damaged the immunity of black carp, in line with findings in grass carp [24]. Taken together, dietary EA reduced the antioxidant and immune capacities in black carp. 

## 5. Conclusions

In summary, our results indicated that a high-dose of EA inhibited growth, induced lipid accumulation, reduced antioxidant and immune capabilities, and led to oxidative damage by suppressing lipid catabolism and increasing lipogenesis. Our findings provide new information for selecting rapeseed oil in farmed carnivorous fish.

## Figures and Tables

**Table 1:** Nutrient composition of the experimental diets.

**Ingredients**	**Dietary Erucic Acid Levels (%)**					
	**0.00**	**0.44**	**0.81**	**1.83**	**2.74**	**3.49**
Casein	36.40	36.40	36.40	36.40	36.40	36.40
Gelatin	9.10	9.10	9.10	9.10	9.10	9.10
Corn starch	32.00	32.00	32.00	32.00	32.00	32.00
Soybean oil	2.00	2.00	2.00	2.00	2.00	2.00
Rapeseed oil	2.00	2.00	2.00	2.00	2.00	2.00
Lecithin high potency	0.50	0.50	0.50	0.50	0.50	0.50
Butylated hydroxytoluene	0.02	0.02	0.02	0.02	0.02	0.02
Mineral premix ^a^	1.50	1.50	1.50	1.50	1.50	1.50
Vitamin premix ^b^	2.00	2.00	2.00	2.00	2.00	2.00
Carboxy methyl cellulose	3.00	3.00	3.00	3.00	3.00	3.00
Choline chloride	0.50	0.50	0.50	0.50	0.50	0.50
Calcium dihydrogen phosphate	1.50	1.50	1.50	1.50	1.50	1.50
Cellulose	5.48	5.48	5.48	5.48	5.48	5.48
Erucic acid ^c^	0.00	0.47	0.94	1.88	2.82	3.50
Palmitic acid ^d^	3.50	3.03	2.56	1.62	0.68	0.00
Attractant	0.50	0.50	0.50	0.50	0.50	0.50
Total	100	100	100	100	100	100
Proximate analysis						
Actual erucic acid	0.00	0.44	0.81	1.83	2.74	3.49
Crude protein (%)	38.91	38.89	39.01	38.94	38.97	39.06
Crude lipid (%)	6.60	6.70	6.60	6.80	6.80	6.70
Moisture (%)	7.05	7.03	6.95	6.93	6.99	7.01

^a^ Mineral premix (g/kg): KCl 28 g, MgSO_4_ · 7H_2_O 100 g, NaH_2_PO_4_ 215 g, KH_2_PO_4_ 100 g, Ca (H_2_PO_4_)_2_ · H_2_O 265 g, CaCO_3_ 105 g, C_6_H_10_CaO_6_ · 5H_2_O 165 g, FeC_6_H_5_O_7_ · 5H_2_O 12 g, ZnSO_4_ · 7H_2_O 4.76 g, MnSO_4_ · H_2_O 1.07 g, AlCl_3_ · 6H_2_O 0.15 g, CuCl_2_ · 2H_2_O 2.4 g, CoCl_2_ · 6H_2_O 1.4 g, KI 0.23 g, α- Cellulose 0.043 g. ^b^ Vitamin premix (g/kg): VA 1.29 g, VC 57.5 g, VE 20 g, VD_3_ 0.63 g, VK_3_ 1.8 g, VB1 7.5 g, VB_2_ 2.63 g, VB_6_ 1 g, VB12 0.15 g, nicotinic acid 5 g, folic acid 0.19 g, inositol 60 g, biotin 0.75 g, calcium pantothenate 5 g, p-aminobenzoic acid 5 g, α-Cellulose 831.56 g. ^c^ The purity of erucic acid (Thermo Fisher, USA) was 90%. ^d^ Amount of palmitic acid added to guarantee equal lipid levels in each experimental group.

**Table 2:** Fatty acid composition (%) of the lipids in six experimental diets.

Fatty Acids	Dietary Erucic Acid Levels (%)					
	0.00	0.44	0.81	1.83	2.74	3.49
C12:0	0.32	0.35	0.40	0.32	0.37	0.29
C14:0	0.94	1.20	1.12	1.14	1.02	0.97
C16:0	60.45	48.68	45.67	25.18	17.98	7.11
C16:1n-7	0.32	0.35	0.33	0.37	0.33	0.33
C18:0	2.58	2.76	2.66	2.96	2.60	2.57
C18:1n-9	15.78	17.90	16.65	19.39	16.65	16.46
C18:2n-6	16.18	18.08	16.90	18.94	16.44	15.76
C18:3n-3	0.18	0.21	0.22	0.30	0.31	0.35
C20:0	3.12	3.76	3.60	4.28	3.89	3.98
C20:1n-9	0.14	0.16	0.14	0.17	0.15	0.14
C22:1n-9	nd	6.56	12.31	26.97	40.27	52.04
Total SFAs	67.41	56.75	53.45	33.87	25.86	14.92
Total MUFAs	16.24	24.96	29.43	46.89	57.39	68.97
Total PUFAs	16.35	18.29	17.12	19.24	16.75	16.10
Total n-3 PUFAs	0.18	0.21	0.22	0.30	0.31	0.35
Total n-6PUFAs	16.18	18.08	16.90	18.94	16.44	15.76

SFAs: saturated fatty acids; MUFAs: monounsaturated fatty acids; PUFAs: polyunsaturated fatty acids; total SFAs include C12:0, C14:0, C16:0, C18:0, C20:0; total MUFAs include C16:1n-7, C18:1n-9, C20:1n-9, C 22:1n-9; total PUFAs include C18:2n-6, C18:3n-3; total n-3 PUFAs include C18:3n-3; total n-6 PUFAs include C18:2n-6; nd: not detected.

**Table 3:** Primer sequences used in this study.

Gene	Sense and Antisense Primer (5′-3′)	Product Length	GenBank NO.	TM (°C)
*cat*	TTGAGCAGGCGGAGAACTGGAA	105	ON987244.1	60
	TTCGGTTCAACACAAGGCGTCC			60
*Mn-sod*	GGTTCGCACCTTCTACACTCA	102	ON987242.1	60
	ACCACCTGTTCACCACGACCAT			60
*Cu-Zn sod*	AAGACACGTCGGAGACCTTGGT	104	ON987241.1	60
	TGATGGAGTCTGGCCCTGACAG			60
*il-1β*	ACCAACACGACCATGCAGTGC	103	ON987262.1	60
	TGCCGTCTTTCAGCGTCATAGC			60
*il-10*	TGGAGACCATTCTGCCAACAGC	105	ON987265.1	60
	TGACCATATCCCGCTTGAGACC			60
*hsl*	CAGACGCCTCACCATCCAGACT	101	MW088569	60
	CTGACAACCCGCACCAGCATAG			60
*aco*	GGCGGCGAATACAGGCATTGA	109	MW053374.1	60
	AGTGCAGGTCGGCGTAAAGGT			60
*cpt1a*	GCAGCGAATTGTTGGTGGTGTG	109	MW048769.1	60
	GAACATCCAGCCATGCCAGGAC			60
*cpt1b*	ACCGGATGGCATCGACCTACAG	101	MW048771.1	60
	TGTTCTTGAAGCGGATGGCACG			60
*pparα*	TGTTCTGTCAGTACGCCTCGGT	108	MK986685.1	60
	TCAGCAGAGTCACCTGGTCGTT			60
*accα*	ACCAGCGAGAACCCAGATGAGG	111	MW053372.1	60
	TCCAGCAGCCGCCACACTAA			60
*fas*	GAGCTGGAACAGACGGTGGAGA	102	MW088571.1	60
	CGTGTCCAAGCAGTGGCGTAAT			60
*srebp-1c*	CCAGGCGGACAACCACATAAGG	115	MW048767.1	60
	TCCCAGCCACCAGGTCTTTGAG			60
*gpat*	GGCTGTGTGCGTGTGGATTTCA	104	MW053377.1	60
	AGGATCTGCTCCAGCGTGAGAG			60
*β-actin*	CCCTGTCCACCTTCCAGCAGAT	110	KP185128.1	60
	CGGCGTGAAGTGGTAACAGTCC			60

The abbreviations used are as follows: *cat*: catalase; *Mn-sod*: Mn-superoxide dismutase; *Cu-Zn sod*: Cu-Zn superoxide dismutase; *il-1β*: interleukin 1 beta; *il-10*: interleukin 10; *hsl*: hormone sensitive lipase; *aco*, acyl-CoA oxidase; *cpt1a*: carnitine palmitoyl transferase 1a; *cpt1b*: carnitine palmitoyl transferase 1b; *pparα*: peroxisome proliferator-activated receptor α; *accα*: acetyl-CoA carboxylase α; *fas*: fatty acid synthase; *srebp-1c*: sterol regulatory element-binding transcription factor 1c; *gpat*: glycerol-3-phosphate acyltransferase.

**Table 4:** Effects of dietary EA on the growth performance, whole body composition and feed utilization of black carp.

Growth Performance	Dietary Erucic Acid Levels (%)					
	0.00	0.44	0.81	1.83	2.74	3.49
IBW (g)	7.78 ± 0.01	7.79 ± 0.01	7.78 ± 0.01	7.76 ± 0.01	7.78 ± 0.01	7.77 ± 0.01
FBW (g)	14.23 ± 0.32 ^ab^	14.69 ± 0.69 ^ab^	15.06 ± 0.22 ^b^	14.47 ± 0.39 ^ab^	13.95 ± 0.16 ^ab^	13.26 ± 0.26 ^a^
WG (%)	83.00 ± 4.02 ^ab^	81.24 ± 2.61 ^ab^	93.53 ± 2.72 ^b^	86.64 ± 5.24 ^ab^	79.32 ± 2.32 ^ab^	70.60 ± 3.41 ^a^
SGR (%/d)	0.83 ± 0.03 ^ab^	0.87 ± 0.06 ^ab^	0.90 ± 0.02 ^b^	0.85 ± 0.04 ^ab^	0.80 ± 0.02 ^ab^	0.73 ± 0.03 ^a^
VSI (%)	6.58 ± 0.18	6.55 ± 0.28	6.26 ± 0.14	6.40 ± 0.21	6.48 ± 0.12	6.25 ± 0.12
HSI (%)	2.33 ± 0.11	2.10 ± 0.10	2.23 ± 0.06	2.27 ± 0.08	2.25 ± 0.07	2.27 ± 0.05
MFI (%)	0.48 ± 0.03	0.46 ± 0.06	0.46 ± 0.05	0.54 ± 0.07	0.40 ± 0.04	0.44 ± 0.04
CF (g/cm^3^)	1.64 ± 0.10 ^a^	1.84 ± 0.04 ^b^	1.84 ± 0.02 ^b^	1.85 ± 0.02 ^b^	1.82 ± 0.02 ^b^	1.82 ± 0.02 ^b^
FCR	2.79 ± 0.16	3.15 ± 0.32	2.52 ± 0.17	2.54 ± 0.13	2.91 ± 0.32	3.41 ± 0.11
Whole body composition						
Moisture (%)	73.08 ± 0.65	72.65 ± 0.43	73.03 ± 0.17	73.22 ± 0.39	73.18 ± 0.49	72.35 ± 0.29
Ash (%)	3.00 ± 0.02	3.07 ± 0.03	2.86 ± 0.11	3.00 ± 0.03	2.97 ± 0.03	2.95 ± 0.09
Lipid (%)	6.34 ± 0.17 ^a^	6.35 ± 0.25 ^a^	6.83 ± 0.10 ^ab^	7.23 ± 0.09 ^b^	7.26 ± 0.09 ^b^	7.38 ± 0.13 ^b^
Protein (%)	17.79 ± 0.27 ^b^	17.86 ± 0.08 ^b^	17.38 ± 0.23 ^ab^	17.15 ± 0.22 ^ab^	16.51 ± 0.24 ^a^	16.51 ± 0.26 ^a^

IBW: initial body weight; FBW: final body weight; WG: weight gain (%) = 100 × (final body weight—initial body weight)/initial body weight; SGR: specific growth rate (%/day) = 100 × [ln (final body weight)—ln (initial body weight)]/days; VSI: viscerosomatic index (%) = viscera weight (g) × 100/fish weight (g); HSI: hepatosomatic index (%) = 100 × liver weight (g)/fish weight (g); MFI: mesenteric fat index (%) = 100 × mesenteric fat weight (g)/fish weight (g); CF: condition factor (g/cm^3^) = fish weight (g) × 100/body length^3^ (cm); FCR: feed conversion ratio = feed intake (g, dry weight)/fish weight gain (g, wet weight). Values displayed in the table are the mean ± SEM (*n* = 12), and means in the same row with different superscripts are significantly different (*p* < 0.05).

**Table 5:** Effects of dietary EA on serum biochemical indicators of black carp.

Parameters	Dietary Erucic Acid Levels (%)					
	0.00	0.44	0.81	1.83	2.74	3.49
Metabolites						
TG (mmol/L)	6.03 ± 0.17 ^a^	6.59 ± 0.13 ^b^	6.89 ± 0.13 ^bc^	6.99 ± 0.13 ^bc^	7.30 ± 0.04 ^c^	7.94 ± 0.09 ^d^
TC (mmol/L)	3.83 ± 0.03 ^a^	3.99 ± 0.06 ^b^	4.16 ± 0.07 ^b^	4.05 ± 0.02 ^b^	3.97 ± 0.26 ^b^	4.92 ± 0.06 ^b^
GLU (mmol/L)	4.53 ± 0.14 ^c^	4.39 ± 0.19 ^c^	3.81 ± 0.08 ^ab^	4.27 ± 0.05 ^b^	2.50 ± 0.08 ^a^	2.84 ± 0.04 ^a^
Antioxidant capacity						
MDA (nmol/mL)	21.58 ± 0.93	19.54 ± 0.95	18.68 ± 0.58	19.14 ± 0.68	20.68 ± 1.05	21.49 ± 0.91
H_2_O_2_ (mmol/L)	172.80 ± 2.49 ^abc^	162.12 ± 2.39 ^a^	168.67 ± 2.30 ^ab^	181.42 ± 2.61 ^bcd^	189.55 ± 2.28 ^d^	184.63 ± 5.18 ^cd^
CAT (U/mL)	9.67 ± 0.87 ^b^	2.35 ± 0.49 ^a^	3.39 ± 1.12 ^a^	4.74 ± 1.17 ^a^	1.41 ± 0.28 ^a^	1.64 ± 0.46 ^a^
Hepatic injury						
AST (U/L)	13.86 ± 2.17 ^a^	21.96 ± 4.73 ^a^	59.79 ± 15.33 ^a^	131.96 ± 11.96 ^b^	201.81 ± 15.66 ^c^	237.87 ± 26.82 ^c^
ALT (U/L)	2.36 ± 0.56 ^a^	5.49 ± 0.77 ^a^	62.96 ± 3.81 ^bc^	56.04 ± 2.10 ^b^	78.32 ± 3.03 ^d^	66.84 ± 2.89 ^c^
Immunocompetence						
ACP (U/L)	49.96 ± 0.77 ^c^	42.53 ± 1.30 ^ab^	40.68 ± 1.11 ^ab^	44.52 ± 1.10 ^b^	39.29 ± 1.01 ^a^	39.39 ± 1.00 ^a^
AKP (U/L)	50.35 ± 0.70 ^b^	42.69 ± 1.34 ^a^	49.22 ± 1.70 ^b^	42.59 ± 1.04 ^a^	39.48 ± 0.99 ^a^	39.54 ± 0.97 ^a^

TG: triglycerides; TC: total cholesterol; GLU: glucose; MDA, malondialdehyde; H_2_O_2_, hydrogen peroxide; CAT, catalase; AST: aspartate aminotransferase; ALT: alanine aminotransferase; ACP: acid phosphatase; AKP: alkaline phosphatase. Values are presented as means ± SEM (*n* = 8), and different superscript letters indicate significant differences (*p* < 0.05).

**Table 6:** Effects of dietary EA on liver biochemical indicators of black carp.

Parameters	Dietary Erucic Acid Levels (%)					
	0.00	0.44	0.81	1.83	2.74	3.49
Lipid metabolites						
Total lipid (%)	8.53 ± 0.14 ^a^	8.37 ± 0.09 ^a^	8.46 ± 0.09 ^a^	8.18 ± 0.21 ^a^	8.81 ± 0.18 ^a^	10.44 ± 0.15 ^b^
TG (mmol/g prot)	0.15 ± 0.00 ^a^	0.17 ± 0.00 ^a^	0.15 ± 0.00 ^a^	0.17 ± 0.00 ^a^	0.24 ± 0.01 ^c^	0.20 ± 0.01 ^b^
TC (mmol/g prot)	0.01 ± 0.00 ^a^	0.02 ± 0.00 ^b^	0.02 ± 0.00 ^b^	0.03 ± 0.00 ^b^	0.03 ± 0.00 ^b^	0.03 ± 0.00 ^b^
Antioxidant capacity						
MDA (nmol/mg prot)	0.90 ± 0.04 ^bc^	0.70 ± 0.02 ^a^	0.82 ± 0.05 ^ab^	0.81 ± 0.05 ^ab^	0.99 ± 0.06 ^bc^	1.07 ± 0.04 ^c^
H_2_O_2_ (mmol/g prot)	9.22 ± 0.23 ^a^	12.67 ± 0.26 ^b^	14.87 ± 0.19 ^c^	15.32 ± 0.21 ^c^	15.85 ± 0.35 ^c^	24.59 ± 0.36 ^d^
T-AOC (U/mg prot)	0.21 ± 0.00 ^c^	0.21 ± 0.00 ^c^	0.18 ± 0.00 ^b^	0.19 ± 0.00 ^b^	0.20 ± 0.00 ^c^	0.15 ± 0.00 ^a^
T-SOD (U/mg prot)	4.46 ± 0.19 ^b^	4.38 ± 0.07 ^b^	4.19 ± 0.05 ^b^	3.69 ± 0.10 ^a^	4.19 ± 0.06 ^b^	4.13 ± 0.11 ^ab^
CAT (U/mg prot)	0.70 ± 0.03 ^b^	0.39 ± 0.02 ^a^	0.33 ± 0.02 ^a^	0.39 ± 0.05 ^a^	0.42 ± 0.02 ^a^	0.42 ± 0.01 ^a^
Immunocompetence						
ACP (U/g prot)	1148.12 ± 42.06 ^b^	1015.62 ± 71.18 ^ab^	1009.52 ± 31.41 ^ab^	1114.43 ± 23.80 ^b^	895.78 ± 44.26 ^a^	979.16 ± 27.57 ^ab^
AKP (U/g prot)	179.70 ± 17.25 ^b^	231.35 ± 14.20 ^c^	205.17 ± 9.53 ^bc^	209.67 ± 5.28 ^bc^	102.75 ± 5.30 ^a^	123.49 ± 6.40 ^a^

TG: triglycerides; TC: total cholesterol; MDA, malondialdehyde; H_2_O_2_, hydrogen peroxide; T-AOC, total antioxidant capacity; T-SOD, total-superoxide dismutase; CAT, catalase; ACP: acid phosphatase; AKP: alkaline phosphatase. Values are presented as means ± SEM (*n* = 8), and different superscript letters indicate significant differences (*p* < 0.05).

**Table 7:** Fatty acid composition (percentages of total fatty acids) in liver of black carp fed with different levels of EA for 8 weeks.

Fatty Acids (%)	Dietary Erucic Acid Levels (%)					
	0.00	0.44	0.81	1.83	2.74	3.49
C14:0	1.35 ± 0.11 ^ab^	1.22 ± 0.03 ^a^	1.45 ± 0.01 ^bc^	1.34 ± 0.02 ^ab^	1.40 ± 0.01 ^bc^	1.54 ± 0.01 ^c^
C16:0	13.85 ± 0.13 ^a^	14.52 ± 0.10 ^b^	14.43 ± 0.22 ^b^	14.23 ± 0.11 ^ab^	15.40 ± 0.06 ^c^	14.08 ± 0.04 ^ab^
C16:1n-7	8.73 ± 0.16 ^a^	8.81 ± 0.09 ^a^	10.80 ± 0.16 ^c^	9.52 ± 0.23 ^b^	9.31 ± 0.03 ^ab^	10.39 ± 0.04 ^c^
C18:0	4.29 ± 0.06 ^d^	3.96 ± 0.02 ^c^	3.82 ± 0.03 ^bc^	3.67 ± 0.07 ^b^	3.39 ± 0.04 ^a^	3.33 ± 0.04 ^a^
C18:1n-9	52.74 ± 0.25 ^c^	49.92 ± 0.31 ^b^	50.21 ± 0.57 ^b^	47.60 ± 0.21 ^a^	47.48 ± 0.15 ^a^	49.48 ± 0.09 ^b^
C18:2n-6	7.07 ± 0.32 ^abc^	7.52 ± 0.08 ^bc^	6.85 ± 0.17 ^bc^	7.44 ± 0.18 ^abc^	7.65 ± 0.03 ^c^	6.77 ± 0.01 ^a^
C18:3n-3	0.06 ± 0.00	0.07 ± 0.00	0.06 ± 0.00	0.06 ± 0.00	0.06 ± 0.00	0.07 ± 0.01
C18:3n-6	0.12 ± 0.00 ^a^	0.12 ± 0.00 ^a^	0.12 ± 0.01 ^a^	0.15 ± 0.01 ^b^	0.20 ± 0.00 ^c^	0.17 ± 0.00 ^bc^
C20:1n-9	4.01 ± 0.12 ^abc^	3.99 ± 0.05 ^bc^	3.80 ± 0.03 ^a^	4.29 ± 0.16 ^bcd^	4.43 ± 0.02 ^d^	4.37 ± 0.01 ^cd^
C20:3n-6	1.35 ± 0.01 ^c^	1.44 ± 0.00 ^d^	1.22 ± 0.02 ^b^	1.38 ± 0.02 ^c^	1.23 ± 0.01 ^c^	1.08 ± 0.00 ^a^
C20:4n-6	1.49 ± 0.01 ^a^	2.81 ± 0.03 ^c^	2.41 ± 0.05 ^b^	3.25 ± 0.11 ^d^	2.80 ± 0.03 ^c^	2.51 ± 0.03 ^b^
C22:0	0.12 ± 0.02 ^c^	0.04 ± 0.00 ^a^	0.05 ± 0.00 ^a^	0.09 ± 0.00 ^b^	0.08 ± 0.00 ^b^	0.07 ± 0.00 ^b^
C22:1n-9	nd	0.30 ± 0.01 ^a^	0.36 ± 0.01 ^a^	0.69 ± 0.07 ^b^	0.89 ± 0.01 ^c^	0.94 ± 0.00 ^c^
C22:5n-3	0.47 ± 0.03 ^a^	0.49 ± 0.02 ^ab^	0.41 ± 0.02 ^a^	0.63 ± 0.03 ^c^	0.60 ± 0.00 ^c^	0.56 ± 0.00 ^bc^
C22:6n-3	4.36 ± 0.01 ^ab^	4.81 ± 0.06 ^bc^	4.04 ± 0.20 ^a^	5.68 ± 0.23 ^d^	5.09 ± 0.05 ^cd^	4.66 ± 0.05 ^bc^
Total SFA	19.61 ± 0.06 ^abc^	19.74 ± 0.11 ^bc^	19.74 ± 0.26 ^bc^	19.33 ± 0.14 ^ab^	20.26 ± 0.09 ^c^	19.03 ± 0.07 ^a^
Total MUFAs	65.48 ± 0.29 ^b^	63.01 ± 0.28 ^a^	65.16 ± 0.70 ^b^	62.09 ± 0.14 ^a^	62.11 ± 0.17 ^a^	65.16 ± 0.13 ^b^
Total PUFAs	14.91 ± 0.35 ^a^	17.25 ± 0.17 ^b^	15.10 ± 0.46 ^a^	18.58 ± 0.25 ^c^	17.63 ± 0.11 ^bc^	15.80 ± 0.07 ^a^
Total n-3 PUFAs	4.88 ± 0.03 ^ab^	5.36 ± 0.07 ^bc^	4.51 ± 0.22 ^a^	6.37 ± 0.24 ^d^	5.74 ± 0.06 ^cd^	5.28 ± 0.05 ^bc^
Total n-6PUFAs	10.02 ± 0.32 ^a^	11.89 ± 0.11 ^b^	10.59 ± 0.24 ^a^	12.21 ± 0.12 ^b^	11.89 ± 0.07 ^a^	10.52 ± 0.02 ^b^

SFAs: saturated fatty acids; MUFAs: monounsaturated fatty acids; PUFAs: polyunsaturated fatty acids; total SFAs include C14:0, C16:0, C18:0, C22:0; total MUFAs include C16:1n-7, C18:1n-9, C20:1n-9, C 22:1n-9; total PUFAs include C18:2n-6, C18:3n-3, C18:3n-6, C20:3n-6, C20:4n-6, C22:5n-3, C20:6n-3; total n-3 PUFAs include C18:3n-3, C22:5n-3, C20:6n-3; total n-6 PUFAs include C18:2n-6, C18:3n-6, C20:3n-6, C20:4n-6; nd: not detected. Values are presented as means ± SEM (n = 8), and different superscript letters indicate significant differences (*p* < 0.05).

## Data Availability

The data presented in this study are available in the main article.
